# Interferon regulatory factor 4/5 signaling impacts on microglial activation after ischemic stroke in mice

**DOI:** 10.1111/ejn.13778

**Published:** 2018-01-16

**Authors:** Abdullah Al Mamun, Anjali Chauhan, Haifu Yu, Yan Xu, Romana Sharmeen, Fudong Liu

**Affiliations:** ^1^ Department of Neurology The University of Texas Health Science Center at Houston McGovern Medical School Houston TX 77030 USA; ^2^ Department of Neurology Shanghai Fengxian District Central Hospital Shanghai China

**Keywords:** IRF4, IRF5, ischemic stroke, microglia, neuroinflammation

## Abstract

Microglial activation is a key element in initiating and perpetuating inflammatory responses to stroke. Interferon regulatory factor 5 (IRF5) and IRF4 signaling have been found critical in mediating macrophage pro‐inflammatory (M1) and anti‐inflammatory (M2) phenotypes, respectively, in peripheral inflammation. We hypothesize that the IRF5/4 regulatory axis also mediates microglial activation after stroke. C57BL6 mice of 8–12 weeks were subject to a 90‐min middle cerebral artery occlusion, and the brains evaluated at 24 h, 3, 10 and 30 days after reperfusion. Flow cytometry was utilized to examine microglial activation and cytokine expression. RT‐PCR was performed for mRNA levels of IRF5/4 in sorted microglia. Microglial expression of IRF5/4 was examined by immunohistochemistry, and brain cytokine levels were determined by ELISA. Our results revealed that the IRF5 mRNA level in sorted microglia increased at 3 days of stroke; whereas IRF4 mRNA level exhibited biphasic increases, with a transient rise at 24 h and a peak at 10 days. The same pattern was seen in IRF5/4 protein colocalization with Iba‐1^+^ cells by IHC. Intracellular levels of TNF‐α and IL‐1β in microglia peaked at 3 days of stroke, and IL‐4^+^ IL‐10^+^ double‐positive microglia significantly increased at day 10. Brain levels of these cytokines were consistent with microglial cytokine changes. Worse behavior test results were seen at 3 days vs. 10 days of stroke. We conclude that microglia phenotypes are dynamic to ischemic stroke, and IRF5/4 signaling may regulate microglial M1/M2 activation and impact on stroke outcomes.

## Introduction

Inflammatory responses play a critical role in the pathophysiology of ischemic stroke (Jin *et al*., 2010; Courties *et al*., 2014b). Brain injury triggers activation of microglia within minutes after ischemia (Pan *et al*., 2015). Once activated, microglia become ‘resident macrophages’ of the brain, in keeping with their derivation from myeloid immune cells (Perry & Teeling, 2013; Fu *et al*., 2014). It is widely accepted that microglia exhibit states of dynamic equilibrium within the ischemic brain as either classically activated M1 or alternatively activated M2 phenotype (Taylor & Sansing, 2013; Hu *et al*., 2015). M1 is a toxic cellular state associated with an increase in protein synthesis of pro‐inflammatory mediators (IFN‐γ, IL1‐β, TNF‐α, IL6, CXCL10, etc.), ROS and NO production, and proteolytic enzymes (MMP 9, MMP3) (Kriegebaum *et al*., 2010; Yona *et al*., 2013). In contrast, M2 microglia release anti‐inflammatory mediators (IL10, TGF‐β, IL4, IL13, IGF‐1) (Zhou *et al*., 2013) and enhance expression of genes associated with inflammation resolution, scavenging and homeostasis as well as basal neurogenesis (Nikolakopoulou *et al*., 2013; Cherry *et al*., 2014; Norden *et al*., 2014). Studies with brain ischemia models reported that M1‐polarized microglia exacerbate neuronal death while the M2 phenotype protects neurons and promotes post‐injury tissue repair (Czeh *et al*., 2011; Jin *et al*., 2014). Several proteins have been identified as markers for the M1 or M2 phenotype, although there is growing recognition that M1/2 phenotypes can overlap. Conventionally, M1 macrophages express high levels of major histocompatibility complex class II (MHC‐II), which is a commonly used marker for M1 phenotype among others including iNOS, TNF‐α, IL1β and IL6 (Schmitt *et al*., 2000; Crain *et al*., 2013; Burke *et al*., 2014; Martinez & Gordon, 2014). On the other hand, CD206, arginase1, Ym1, IL4, IL10, IL13 and TGFβ are frequently used as specific markers for M2 activation (Crain *et al*., 2013; Martinez & Gordon, 2014; Turtzo *et al*., 2014; Roszer, 2015).

Emerging data showed that members of the interferon regulatory factor (IRF) family are an integral component of the macrophage activation (Gunthner & Anders, 2013). Studies of peripheral inflammation suggested that IRF5 and IRF4 are critical for M1 and M2 macrophage polarization, respectively (Satoh *et al*., 2010; Masuda *et al*., 2014). IRF‐5 is required for TLR‐mediated expression of IL‐6, TNF, IL‐12 and other pro‐inflammatory cytokines in macrophage, leading to M1 phenotype (Gunthner & Anders, 2013). In contrast, IRF4 was identified as a key transcription factor for controlling M2 macrophage priming and polarization (Honma *et al*., 2005; Gunthner & Anders, 2013). *In vivo* silencing of IRF5 reprogrammes the macrophage phenotype toward M2 polarization and improves infarct healing in cardiac ischemia models (Courties *et al*., 2014a). On the contrary, IRF4^−/−^ mice are more sensitive to LPS‐induced sepsis and exhibit higher production of pro‐inflammatory cytokines such as TNFα and IL‐6 as well as decreased expression of M2 marker genes such as Arg1, Ym1 and Fizz1 (Honma *et al*., 2005; Satoh *et al*., 2010). Whether IRF5 and IRF4 also mediate microglial activation after stroke is not known. In this study, we examined the IRF5/4 signaling in ischemic microglia and evaluated the impacts of the two IRFs on post‐stroke inflammation and outcomes.

## Materials and Methods

### Experimental animals

All animal protocols were approved by the University's Institutional Animal Care and Use Committee and were performed in accordance with National Institutes of Health and University of Texas Health Science Center at Houston (UTHealth) animal guidelines. Young (8–12 weeks) C57BL6 male mice were from the Jackson Laboratory and acclimated to the housing conditions in an ambient temperature and humidity controlled vivarium, with a 12‐ to 12‐h day‐night cycle and free access to food and water *ad libitum*.

### Ischemic stroke model

Cerebral ischemia was induced by 90‐min reversible middle cerebral artery occlusion (MCAO, 20–25 gm mice) under isoflurane anesthesia as previously described (Liu *et al*., 2009). Rectal temperatures were maintained at approximately 37 °C during surgery and ischemia with an automated temperature control feedback system. A midline ventral neck incision was made, and unilateral MCAO was performed by inserting a 6.0‐mm monofilament (Doccol Corp, Redlands, CA, USA) into the right internal carotid artery 6 mm from the internal carotid/pterygopalatine artery bifurcation via an external carotid artery stump. Regional cerebral blood flow was measured in all stroke animals using laser Doppler flowmetry. Animals that showed a regional cerebral blood flow reduction by at least 85% from baseline levels during MCAO were included for further experimentation. After reperfusion, mice were killed at 24 h and 3, 10 and 30 days depending on experimental designs. Sham‐operated animals underwent the same surgical procedure, but the suture was not advanced to the middle cerebral artery. Total of 147 mice (55 sham‐operated and 92 ischemic mice) were used in this study, including eight mice that were excluded from further assessments because of either death after ischemia or failure in ischemia induction. Among them, a number of six stroke/four sham animals per group were used for each experiment.

### Immunohistochemistry

**Figure 1:**
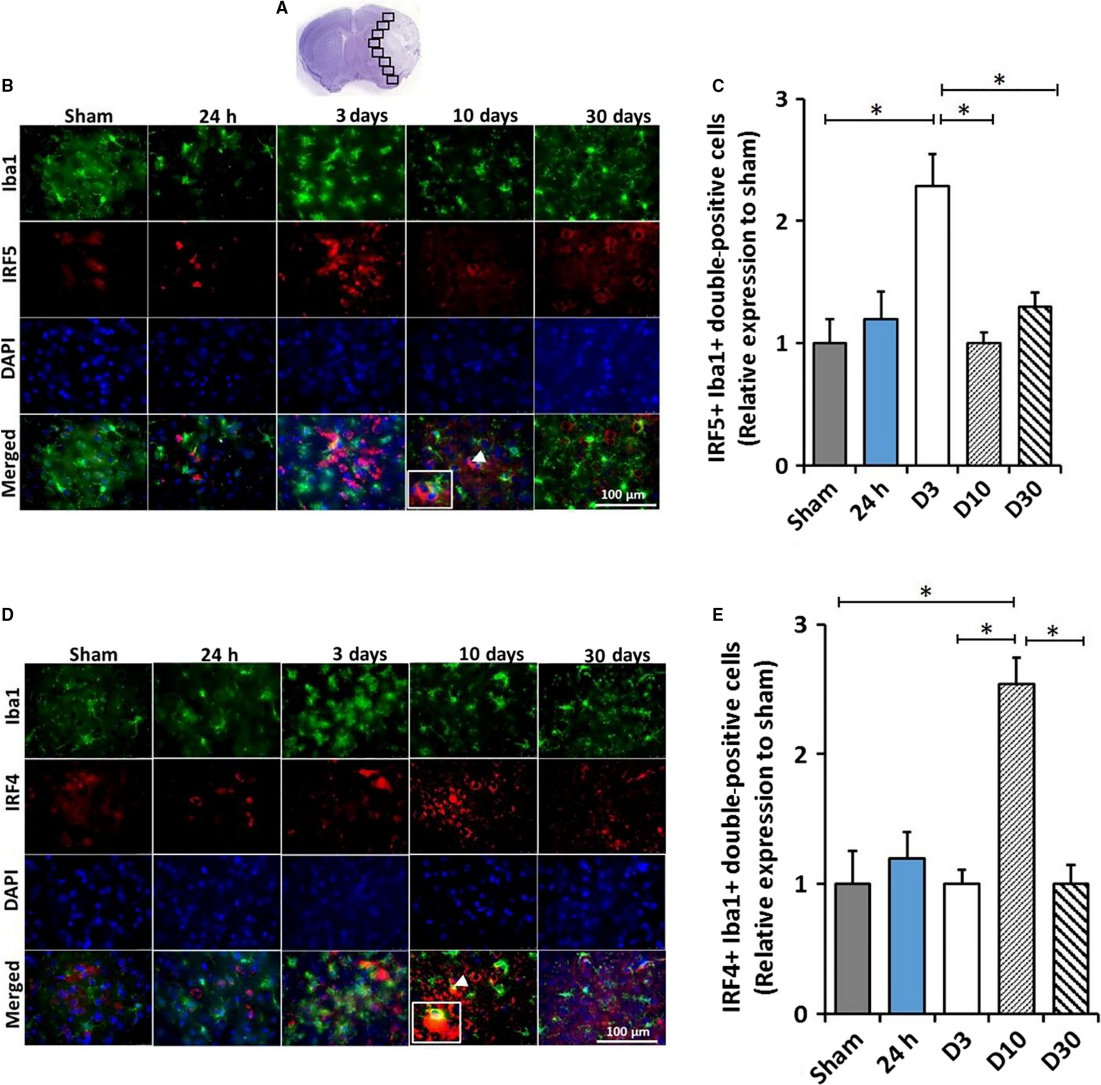
IRF5/4 expression in activated microglia/macrophages at 24 h, 3, 10 and 30 days following 90‐min MCAO. Image analysis was performed in eight ipsilateral regions (black boxes) at the inner boundary zone of the infarct (A). Co‐expression of IRF5 or IRF4 (red) with Iba1 (green) in activated microglia/macrophages was shown in Figure (B) and (D), respectively. IRF5^+^Iba1^+^ and IRF4^+^Iba1^+^ (inset in the d10 merged) double‐positive cells in the peri‐infarct area of the ipsilateral hemisphere of stroke mice were quantified and showed in (C) and (E), respectively. White arrowheads indicate the double‐positive cell as shown in the inset box. All the quantification data were presented as relative changes compared to sham as fold change. *N *= 6 stroke/4 sham animals per group; *P <* *0.05. [Colour figure can be viewed at http://wileyonlinelibrary.com].

The mice were anesthetized by tribromoethanol (Avertin^®^) ip injection at a dose of 0.25 mg/g of body weight, and then transcardially perfused with 0.1M sodium phosphate buffer (pH 7.4) followed by 4% paraformaldehyde for post‐fixation of the brains. Immunohistochemical staining of fixed‐frozen sections (30 μm thickness) was performed as described previously (Liu *et al*., 2009). Briefly, the brains were cut and mounted onto gelatin‐coated slides and allowed to air‐dry. The sections were then blocked in 0.1M PBS with 0.25% Triton X‐100 (Sigma) and 10% donkey serum for 2 hours and incubated overnight at 4 °C with the following primary antibodies: rabbit anti‐Iba1 (1:300, Wako, Japan), goat anti‐IRF4 (sc6059; 1 : 50, Santa Cruz) and goat anti‐IRF5 (AP16060PU‐T; 1 : 50, OriGene Technologies). After being washed in TBS+0.05% Tween 20, the sections were incubated with the indicated secondary antibodies for 1 h. The following secondary antibodies were used: donkey rabbit anti‐mouse IgG Alexa Fluor 488 conjugate (A21206; 1: 500; Invitrogen, USA) and donkey anti‐goat IgG Alexa Fluor 594 conjugate (A11011; 1: 500; Invitrogen). The nuclei were stained with DAPI (S36939; Invitrogen). Brain slices were taken at the same distance from bregma to ensure comparison of similar structures. Eight 20× fields/animal were analyzed in the peri‐infarct area at the inner boundary zone of the infarct (Fig. 1A). Double‐positive cells were counted by an unbiased, blinded investigator using ImageJ software (NIH), and the cell numbers were normalized to sham groups.

### Tissue harvesting and flow cytometry

Mice were euthanized by tribromoethanol (Avertin^®^ ip injection at a dose of 0.25 mg/g body weight), transcardially perfused with 60‐mL cold, sterile PBS. After the brains were harvested, the brainstem, cerebellum and olfactory bulbs were removed. The brain was then divided along the interhemispheric fissure into two hemispheres. Ipsilateral brains were placed in complete Roswell Park Memorial Institute (RPMI) 1640 (Lonza) medium, followed by mechanical and enzymatical digestion with 150 μL collagenase/dispase (1 mg/mL) and 300 μL DNAse (10 mg/mL; both Roche Diagnostics) for 45 min at 37 °C with mild agitation. The cell suspension was filtered through a 70‐μm filter. Leukocytes were harvested from the interphase of a 70%/30% Percoll gradient. Cells were washed and blocked with mouse Fc Block (eBioscience) prior to staining with primary antibody‐conjugated fluorophores: CD45‐eF450, CD11b‐AF488, Ly6C‐APC‐eF780, Ly6G‐PE, MHC‐II‐APC and CD206‐PE‐cy5.5. For all of the surface markers, 0.25 μg (1:100) of antibody was used to stain 1 × 10^6^ cells. All the antibodies were commercially purchased from eBioscience. For live/dead discrimination, a fixable viability dye, carboxylic acid succinimidyl ester (CASE‐AF350; Invitrogen), was diluted at 1:300 from a working stock of 0.3 mg/mL. Cells were briefly fixed in 2% paraformaldehyde (PFA). Data were acquired on a CytoFLEX (Beckman Coulter) and analyzed using FlowJo (Treestar Inc.). No <100 000 events were recorded for each sample. For microglia sorting, BD FACS ARIA II was used. Cell type‐matched fluorescence minus one (FMO) controls were used to determine the positivity of each antibody (Fig. S1).

### mRNA extraction and gene expression analysis by real‐time PCR

Total RNA was isolated from sorted microglia using RNAqueous^®^‐Micro Total RNA Isolation Kit (Thermo Fisher Scientific, USA), and cDNA was then synthesized from 30 ng of total RNA with the Sensiscript RT Kit (Qiagen GmbH, Hilden, Germany) in accordance with the manufacturer's instructions. Real‐time polymerase chain reaction (RT‐PCR) was performed with a QuantiTect SYBR Green PCR Kit (Qiagen GmbH) using the BIO‐RAD C1000 Thermal Cycler (*BIO‐RAD,* Hercules, CA, USA). Results were normalized using the housekeeping gene GAPDH and the 2^−ΔΔCt^ cycle threshold method. The primer sequences are as follows:

Forward IRF4: 5′‐CAAAGCACAGAGTCACCTGG‐3′
Reverse IRF4: 5′‐CTGCCCTGTCAGAGTATTTC‐3′
Forward IRF5: 5′‐ CCTCAGCCGTACAAGATCTACGA‐3′
Reverse IRF5: 5′‐ GTAGCATTCTCTGGAGCTCTTCCT‐3′
Forward GAPDH: 5′‐GTGTTCCTACCCCCAATGTGT‐3′
Reverse GAPDH: 5′ ATTGTCATACCAGGAAATGAGCTT‐3′

### Intracellular cytokine staining

For intracellular cytokine staining, an ex vivo brefeldin A protocol was followed. Prior to staining, brain leukocytes were incubated with BFA (10 μg/mL, Sigma) in 1 mL complete RPMI for 2 h at 37 °C (5% CO_2_). Afterward, cells were resuspended in Fc Block, stained for surface antigens and washed in 100 μL of fixation/permeabilization solution (BD Biosciences) for 20 min. Cells were then washed twice in 300 μL permeabilization/wash buffer (BD Biosciences), resuspended in an intracellular antibody cocktail (0.25 μg for each antibody, 1:100 dilution) containing TNFα‐PE‐Cy7 (eBioscience) and IL‐1β‐PE (eBioscience), IL‐10‐APC and IL‐4‐PerCP‐Cy5.5 (BioLegend) and subsequently fixed.

### Brain cytokine levels by ELISA

Ipsilateral brain was homogenized using Dounce Homogenizer in 10 volumes of NP40 cell lysis buffer (FNN0021; Thermo Fisher Scientific) supplemented with 1 mm phenylmethylsulfonyl fluoride (PMSF) and a protease inhibitor cocktail (Sigma‐Aldrich). All steps were carried out at 4 °C. The homogenate was centrifuged initially at 700 ***g*** for 5 min to eliminate unruptured cells and debris and then further centrifuged at 12 500 ***g*** for 20 min, and the supernatant was used to measure cytokine levels by ELISA. Tumor necrosis factor‐alpha (TNF‐α), IL‐1β, interleukin‐10 (IL‐10) and IL‐4 levels were measured by commercially available specific quantitative sandwich ELISA kits according to the manufacturer's instructions (eBioscience). The cytokine levels were normalized by total protein.

### Behavioral assessment

#### Neurological deficit scores

Neurological deficit scores (NDS) were recorded by a 4‐point scale: 0—no deficit; 1—forelimb weakness, torso turning to the ipsilateral side when held by the tail; 2—circling to the affected side; 3—unable to bear weight on affected side and 4—no spontaneous activity or barrel rolling.

#### Corner test

Sensorimotor activity was measured by corner test as described previously (Li *et al*., 2004). Briefly, the mouse entered a corner that was made by moving two cardboard pieces at an angle of 30 degrees in front of the nose. Contact with the vibrissae led to a rear and the direction in which the mouse turned was recorded. Normal mice do not exhibit a turning preference; but after ischemia, mice have a turning preference to the non‐impaired side. The percentage of right turns was calculated for twenty trials for each mouse. The corner test has been used to detect both sensory and motor abnormalities in the mouse stroke model (Li *et al*., 2004; Manwani *et al*., 2011).

#### Wire‐hanging test

The wire‐hanging test was used to evaluate the motor function and deficit in stroked mice as described previously with slight modification (Ji *et al*., 2009). A wire cage top (18 inch × 9 inch) with its edges taped off was used for this experiment. The mouse was placed on the center of the wire lid, and the lid was slowly inverted and placed on top of cage. The wire lid was 9 inch above the cage bedding. Latency to fall from the wire was recorded. The time‐out period was 90 s. All behavioral tests were performed by a blinded investigator.

### Statistical analysis

Data from individual experiments are presented as mean ± SD and assessed by Student's *t*‐test or one‐way ANOVA with Tukey's post hoc test for multiple comparisons (GraphPad Prism Software Inc, San Diego, CA, USA) except NDS, which was analyzed with the Mann–Whitney *U*‐test. Only behavior data from corner test was analyzed by two‐way ANOVA, and significant differences between paired comparisons were conducted with the Holm–Sidak test. Significance was set at *P *< 0.05.

## Results

### Expression of IRF4/5 in Iba‐1^+^ cells in ischemic brains

To examine IRF5/4 expression in microglia after MCAO, we performed IHC and quantified IRF5/4 colocalization with Iba‐1 at 24 h, 3, 10 and 30 days of stroke. The number of double‐positive cells (IRF5^+^Iba‐1^+^ or IRF4^+^Iba‐1^+^) after stroke was normalized to sham groups. The result showed that the fold increase of IRF5^+^Iba‐1^+^ cells was significantly higher at 3 days of stroke and subsequently decreased to the baseline at 10 days (Fig. 1B,C). In contrast, the IRF4^+^Iba‐1^+^ cell number did not increase until 10 days compared to the sham (Fig. 1D,E).

### IRF4/5 mRNA levels in sorted microglia

**Figure 2:**
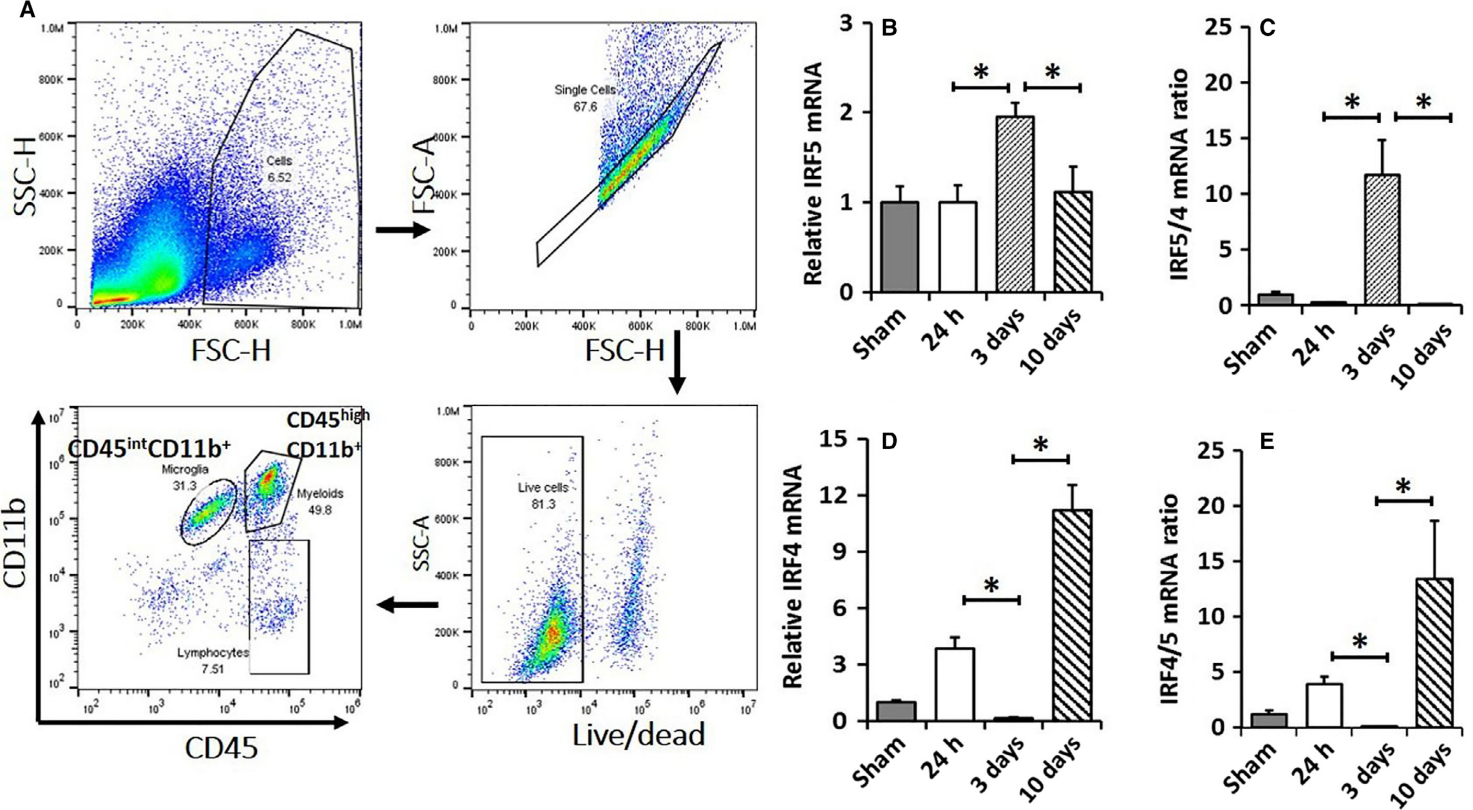
IRF5/4 mRNA levels in activated microglia. A representative dot plot depicts the gating strategy used to sort microglia (A). CD45^int^ CD11b^+^ cells were considered as microglia. Results of Q‐PCR for mRNA expression were shown in (B) for IRF5 and in (D) for IRF4. mRNA ratios of IRF5/IRF4 and IRF4/IRF5 were shown in (C) and (E), respectively. *N *= 6 stroke/4 sham animals per group; *P <* *0.05. [Colour figure can be viewed at http://wileyonlinelibrary.com].

Iba‐1 staining by IHC in ischemic brain slices cannot distinguish microglia from infiltrating monocytes. To confirm the production of IRF5/4 by microglia, we performed cell sorting with flow cytometry and examined the mRNA levels of IRF5/4 in sorted microglia by RT‐PCR. The gating strategy is shown in Fig. 2A. The IRF5 mRNA level was significantly increased at 3 days after stroke and declined to the baseline by day 10 (Fig. 2B). Interestingly, the mRNA level of IRF4 exhibited biphasic increases after stroke. At 24 h of stroke, IRF4 mRNA significantly increased followed by a significantly lower level at day 3 compared to the sham group; at day 10, IRF4 mRNA level skyscraped and was significantly higher than any other group (Fig. 2D). To compare the predominance of IRF5 or IRF4 transcription in microglia, we quantified the mRNA ratio of the two IRFs at different time points. IRF5/4 mRNA ratio also significantly increased at 3 days of stroke (Fig. 2C); IRF4/5 mRNA ratio showed a trend of transient increase at 24 h which significantly decreased at 3 days and significantly increased at 10 days after stroke (Fig. 2E). The data of IRF5/4 mRNA levels were consistent with the pattern of IRF5 and IRF4 expression by IHC in Fig. 1, suggesting microglial expression of IRF5/4 is in a time–course manner and dependent on the progression of stroke. In addition, a high expression of IRF5 was corresponding to a low expression of IRF4 and vice versa, suggesting an interaction between IRF5 and IRF4 signaling after ischemia.

### MHC‐II and CD206 were differentially expressed in microglia

**Figure 3:**
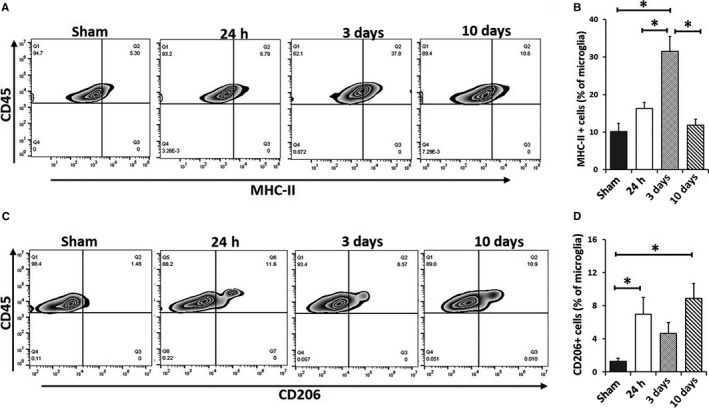
Microglia activation after 90‐min MCAO. Quantification of MHC‐II ^+^ microglia (A, B) and CD206^+^ microglia percentage (C, D) of total gated microglia at 24 h, 3 and 10 days after MCAO. *N *= 6 stroke/4 sham animals per group; *P <* *0.05.

Microglial activation is a key element in initiating and perpetuating inflammatory responses to ischemia (Patel *et al*., 2013; Chen *et al*., 2014). To examine the relationship between IRF5/4 expression and M1/2 microglial activation, we next examined microglial polarization after stroke with flow cytometry. MHC‐II and CD206 are well‐established markers for M1 and M2 microglial activation, respectively (Mirza *et al*., 2015; Pan *et al*., 2015; McCullough *et al*., 2016). We gated microglia with CD45^int^ and CD11b^+^, and then quantified the percentage of MHC‐II^+^ or CD206^+^ microglia to total microglia. Three days after stroke, the percentage of MHC‐II^+^ microglia (M1 microglia) significantly increased compared to the sham and 24 h group (Fig. 3A,B). By day 10, the percentage of MHC‐II^+^ microglia returned to the basal level (Fig. 3B). The percentage of CD206^+^ microglia (M2 microglia) showed a different pattern and significantly increased at 24 h and peaked at 10 days after stroke compared to the sham (Fig. 3C,D). The data of MHC‐II and CD206 indicate that M1/2 microglial polarization is corresponding to IRF5/4 expression.

### Cytokine production by microglia after stroke

**Figure 4:**
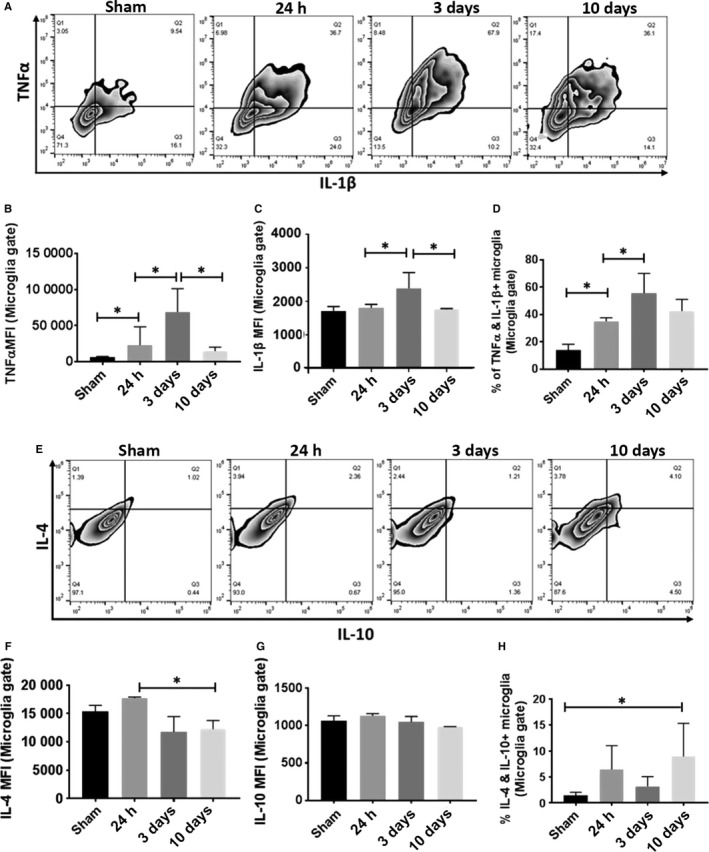
Measurement of intracellular pro‐ and anti‐inflammatory cytokines in microglia at 24 h, 3 and 10 days following 90‐min MCAO. Representative zebra plots (A) and (E) showed microglial production of pro‐inflammatory TNF‐α/IL‐1β and anti‐inflammatory IL‐4/IL‐10, respectively. The expression levels of TNF‐α, IL‐1β, IL‐4 and IL‐10 were measured as mean fluorescence intensity (MFI) in (B), (C), (F) and (G), respectively. Quantification of TNF‐α^+^ IL‐1β^+^ and IL‐4^+^ IL‐10^+^ double‐positive microglia was showed in (D) and (H), respectively. *N *= 8 stroke/5 sham animals per group; *P <* *0.05.

To confirm the time–course change in microglial phenotypes after stroke, we performed both pro‐ and anti‐inflammatory intracellular cytokine staining in microglia (CD45intCD11b+) by flow cytometry. Microglial levels of pro‐inflammatory cytokines (TNFα and IL‐1β) increased after stroke and peaked at day 3, and then significantly decreased at day 10 (Fig. 4A–C). Of note, TNF‐α and IL‐1β double‐positive microglia also significantly increased at 24 h and peaked at 3 days of stroke, which subsequently decreased at day 10 (Fig. 4D). On the other hand, the level of IL‐4 transiently increased at 24 h and then significantly decreased at 3 and 10 days after stroke; no change in IL‐10 levels was observed at each specific time points (Fig. 4E–G). Interestingly, the number of IL‐4 and IL‐10 double‐positive cells significantly increased only at 10 days (Fig. 4H). The intracellular cytokine expression data showed similar pattern as that of microglial IRF5/4 expression (Figs 1 and 2), suggesting that the production of pro‐/anti‐inflammatory cytokines in microglia is related to IRF5/4 signaling, respectively.

### Time–course of brain cytokine levels after stroke

**Figure 5:**
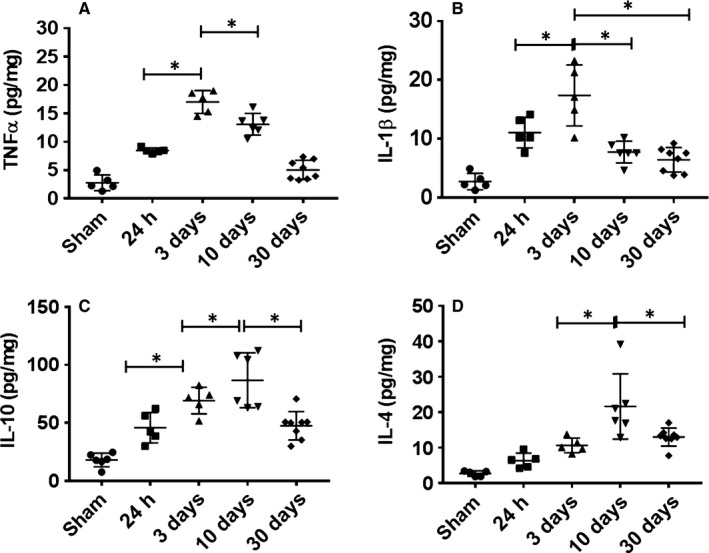
Cytokine levels in the ischemic brain. Pro‐inflammatory (TNFα and IL‐1β) (A and B) and anti‐inflammatory (IL‐10 and IL‐4) (C and D) cytokine levels (pg/mg of protein) were measured in the ipsilateral hemispheres of sham and stroke mice at 24 h, 3, 10 and 30 days after reperfusion. *N *= 6 stroke/4 sham animals per group; *P <* *0.05.

Brain cytokine levels are reflective of the level of immune responses to ischemic injury (Plata‐Salaman, 2002; Minami *et al*., 2006). To investigate whether brain cytokine levels also change following the time–course of IRF5/4 expression, we measured IL‐1β, TNF‐α, IL‐10 and IL‐4 levels in ischemic brains at 24 h, 3, 10 and 30 days. Intriguingly, these pro‐ and anti‐inflammatory cytokines also showed a similar expression pattern as that of IRF5/4. The pro‐inflammatory cytokine (TNFα and IL‐1β) levels were significantly increased at 24 h, 3 and 10 days of stroke, with a peak at day 3 (Fig. 5A,B). In contrast, the levels of anti‐inflammatory cytokines (IL‐4 and IL‐10) increased and peaked at 10 days of stroke (Fig. 5C,D). Again, pro‐inflammatory and anti‐inflammatory responses in the ischemic brain were corresponding to microglial IRF5/4 expression and M1/2 microglial polarization.

### Behavior outcomes correlate with the inflammatory profile of the ischemic brain

**Figure 6:**
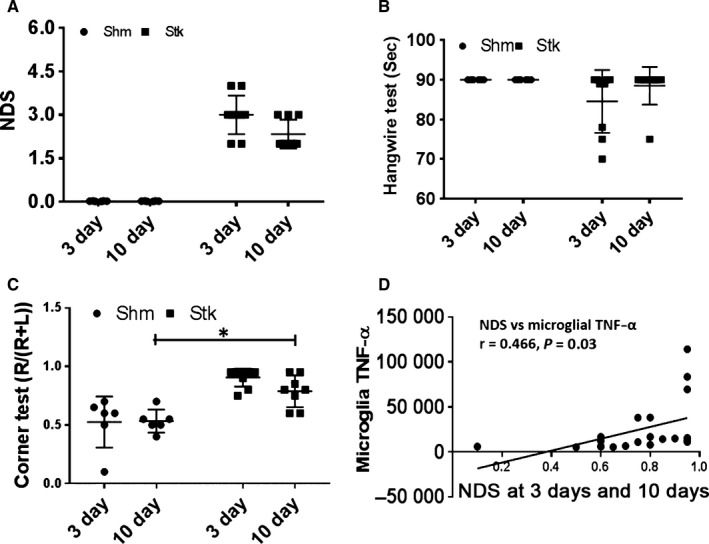
Assessment of behavior outcomes at 3 and 10 days following 90‐min MCAO. Neurological deficit score (NDS) (A), hangwire (B) and corner test (C) were assessed for each group at 3 and 10 days. Microglial intracellular TNF‐α significantly correlated with at 3 and 10 days after stroke (D). *N *= 6–8 animals per group; *P <* *0.05.

To understand the contributing role of inflammatory microenvironment in stroke outcomes, we performed functional behavior analysis including NDS, hangwire and corner test at day 3 and 10. The result showed the NDS significantly decreased at 10 days compared to that of 3 days group (Fig. 6A). In corner test, the proportion of right turn was significantly less at 10 days vs. 3 days (Fig. 6C). There was no time–course difference in the result of hangwire test (Fig. 6B). Interestingly, microglial intracellular pro‐inflammatory cytokine TNF‐α significantly correlated with NDS at 3 and 10 days after stroke (Fig. 6D). The behavior test data suggest that the inflammatory state in the ischemic brain has an impact on stroke outcomes.

## Discussion

The identification of specific mediators of microglial activation may provide a therapeutic target to alleviate microglia‐mediated injury in cerebral ischemia (Lalancette‐Hebert *et al*., 2012; Taylor & Sansing, 2013). In this study, we utilized a widely accepted animal stroke model (i.e., MCAO) and investigated the relationship of IRF5/4 signaling with microglial M1/M2 phenotype switch, and their correlation with stroke outcomes. Several new findings were revealed. Firstly, IRF5 expression correlates with microglia M1 polarization and plays an important role in maintaining a pro‐inflammatory microenvironment in the ischemic brain at the early stage of stroke. Secondly, IRF4 expression corresponds to M2 microglial polarization, which is consistent with the anti‐inflammatory state at the late phase of stroke. Thirdly, IRF5 or IRF4 has an inhibitory effect on each other. The expression of the two IRFs ‘waxed and waned’ with the progress of the disease, suggesting a functional interaction between the two IRFs upon ischemic insults. Finally, there is a time–course change in the inflammatory states in the ischemic brain as the infarct evolves. The inflammatory states significantly affect functional outcomes, with a pro‐inflammatory bond to worse outcomes and an anti‐inflammatory bond to better functional recovery. To our knowledge, this is the first study that examined the IRF5/4 regulatory axis in the context of ischemic stroke and highlighted the roles of IRF5/4 in mediating microglial phenotypic changes after ischemia.

Microglia play a central role in mediating post‐stroke inflammation (Patel *et al*., 2013; Welser‐Alves & Milner, 2013). Previous studies have demonstrated that microglia are the major source of inflammatory cytokines in neuroinflammation (Hanisch, 2002; Welser‐Alves & Milner, 2013). Our data are consistent with these studies as we found the time–course of microglial intracellular cytokine levels exhibited a similar pattern to that of brain total cytokine levels (Figs 4 and 5). Microglial activation is stimulus‐driven and exhibits either pro‐ or anti‐inflammatory phenotype in the ischemic brain (Hu *et al*., 2012, 2015) depending on the molecular signals microglia sense. Once activated, microglia develop macrophage‐like capabilities that have many disparate effects, including cytokine production, antigen presentation, phagocytosis and promotion of tissue repair (Patel *et al*., 2013). The present study found microglia took on an early and transient M2 phenotype at 24 h of stroke (Fig. 3), which is consistent with a previous study (Hu *et al*., 2012), suggesting an innate mechanism to restrict tissue damage; however, the sick microglial M1 phenotype soon predominates, reflecting the cytotoxic stimuli of harmful metabolites released from the ischemic tissue. In our MCAO model, the post‐stroke M2 microglial response was at a low level and did not prevail until 10 days after stroke, suggesting that although the immune response exerts additional damage to brain tissue at the early stage of ischemia, the innate protective immune mechanism (M2) keeps ‘fighting’ and eventually outweighs the pro‐inflammatory response (M1) to conduct tissue repair. The expression of both IRFs and brain cytokine levels returned to the baseline at 30 days of stroke, indicating the inflammatory response in the ischemic brain is quenched at the chronic stage and maintained at a low level. The time–course of microglial M1/2 polarization seems to be fine‐tuned by endogenous inflammatory signaling pathways, highlighting an important role of IRF5/4 signaling in post‐stroke inflammation.

Previous studies have highlighted the importance of IRFs in macrophage phenotype modulation (Lawrence & Natoli, 2011; Wang *et al*., 2014). Different subtype of IRFs exerts a distinct role in macrophage polarization, for example, IRF5 regulates expression of pro‐inflammatory cytokines (TNFα, IL‐1β, etc.) leading to M1 phenotype, whereas IRF4 is a key transcription factor for expression of anti‐inflammatory cytokines (IL‐4, IL‐10, etc.) in macrophages (M2) (Klein *et al*., 2006). Consistent with peripheral inflammatory studies (Satoh *et al*., 2010; Masuda *et al*., 2014), our data suggested that microglial M1 and M2 phenotypes are also regulated by IRF5 and IRF4, respectively. Emerging data have demonstrated the IRF5/4 signaling was implicated in neurodegenerative (Zhu *et al*., 2016; Song *et al*., 2017) and infectious (Takaoka *et al*., 2005; Balkhi *et al*., 2010; Satoh *et al*., 2010; Xuan *et al*., 2016) diseases, and indicated that IRF5/4 are the key determinants for macrophage polarization upon inflammatory stimuli (Al Mamun & Liu, 2017). The present study found the expression of IRF5/4 in ischemic microglia is up‐regulated in a time‐dependent manner that is closely correlated with M1/2 phenotype, respectively, suggesting microglia share the same mechanism as that in peripheral monocytes to respond to self‐antigens. Our study not only found a link between IRF5/4 and M1/2 microglial activation, respectively, but also revealed that IRF5/4 expression accommodates each other. At each time point after ischemia, mRNA levels of the two IRFs waxed and waned in a ‘complementary’ manner (Fig. 2), and the protein expression by IHC also exhibited the similar pattern (Fig. 1), strongly suggesting a regulatory role of IRF5/4 axis in microglial activation. IRF4 has been found to compete with the pro‐inflammatory IRF5 for binding to the adaptor MyD88 that transmits TLR outside‐in signaling for transcription of pro‐inflammatory cytokines (Eguchi *et al*., 2013; Zhao *et al*., 2015). As a result, the competitive action of IRF4 for MyD88 binding renders IRF4 an endogenous TLR signaling antagonist that can suppress M1 macrophage polarization (Takaoka *et al*., 2005, 2008). Similarly, IRF5 not only controls M1 macrophage polarization by promoting the expression of pro‐inflammatory cytokines, but also suppresses the anti‐inflammatory cytokine IL‐10 (Krausgruber *et al*., 2011). It is possible that IRF5/4 regulatory axis balances the pro‐ and anti‐inflammatory pathways and keeps microglia/macrophages in a quiescent ‘M0’ phenotype under normal conditions. However, when inflammation occurs, the balance is lost by ‘aberrant’ expression of IRF5/4 that synergistically signals to induce the M1 or M2 phenotype, which resultantly impacts on stroke outcomes as shown by our behavior data (Fig. 6). It is likely that IRF5 and IRF4 mediated M1 and M2 microglial activation pathways in a ‘co‐operative’ way through ‘conjunction’: IRF5/4 interaction (Zhao *et al*., 2015), which has been reflected by our data in Fig. 2C,E.

The present study has limitations that should be kept in mind when interpreting the data. We found IRF5/4 expression correlates with microglial M1/2 polarization; however, whether IRF5/4 is needed for microglial activation was not tested. Our next step is to manipulate IRF5/4 expression either by overexpression or gene knockout for the mechanistic study. IRF5/4 signaling also functions in infiltrating monocytes; the present study was focused on microglia that have a central role in post‐stroke inflammation. Ongoing studies in the laboratory are investigating contributions of IRF5/4 signaling from microglia and/or infiltrating monocytes on stroke outcome with IRF4/5 conditional knockout (CKO) mice and bone marrow chimera mouse models. We recognize that microglial M1 or M2 activation is not an ‘all‐or‐none’ process, but rather a continuum depending on encountered stimuli (Giunti *et al*., 2014). Therefore, M1 or M2 phenotype is not ‘pure’ but rather reflects the predominant inflammatory state of microglia after an ischemic insult. IRF5/4 regulatory axis may regulate the dynamic balance between M1 and M2 microglial states, and the two phenotypes evolve and transition into each other during the disease progression (Giunti *et al*., 2014; Choi *et al*., 2017).

In conclusion, this study demonstrates the time–course interaction between IRF5/4 and M1/2 microglial phenotypes and provides a new insight into the mechanisms underlying the phenotypic shifts of microglia related to IRF5/4 regulatory axis in response to stroke injury. As the primary element of immune responses to stroke, microglial activation has both beneficial and detrimental effects on outcomes. Future studies aiming at manipulation of the IRF5/4 regulatory axis are warranted to limit ischemic injury and/or promote tissue repair by intervening in microglial M1/2 polarization. The time–course relationship between IRF5/4 expression and M1/2 microglial activation revealed in the present study is critical for future mechanistic studies as it provides the optimal time points for intervention of M1/2 activation states.

## Conflict of interest

All authors do not have conflict of interests.

## Author contributions

AAM participated in the design of the study, performed the experiments, analyzed data and drafted the manuscript. AC and YH participated in the experiment of flow cytometry. YX and RS performed IHC and maintained animal colony and sample collection. FL conceived the study, participated in its design and edited the manuscript. All authors have read and approved the final version of manuscript.

## Data accessibility

This manuscript contained and presented all the datasets that are responsible for the results and conclusions. The authors confirm that all data underlying the findings are available without restriction and will be shared with the research community upon request.

**Abbreviations**
BFA: brefeldin A
CD: cluster of differentiation
IFN‐γ: Interferon‐γ
IL: interleukin
IRF: interferon regulatory factor
MCAO: middle cerebral artery occlusion
MHC‐II: major histocompatibility complex II
MMP: matrix metalloproteinase
NDS: neurological deficit score
PFA: paraformaldehyde
PMSF: phenylmethylsulfonyl fluoride
ROS: reactive oxygen species
RPMI: Roswell Park Memorial Institute
TLR: toll‐like receptor
TNF‐α: tumor necrosis factor‐alpha

## Supporting information

Fig. S1 Cell type‐matched fluorescence minus one (FMO) controls for MHC‐II, TNF, IL‐4, CD206, IL‐1, and IL‐10. The boxed areas indicate the positive zone.
