# Supplementary figures and images for: Interferon regulatory factor 4/5 signaling impacts on microglial activation after ischemic stroke in mice
Source: Eur J Neurosci. 2018 Jan 16;47(2):140–9. doi: 10.1111/ejn.13778 (PMC5771847; doi:10.1111/ejn.13778)

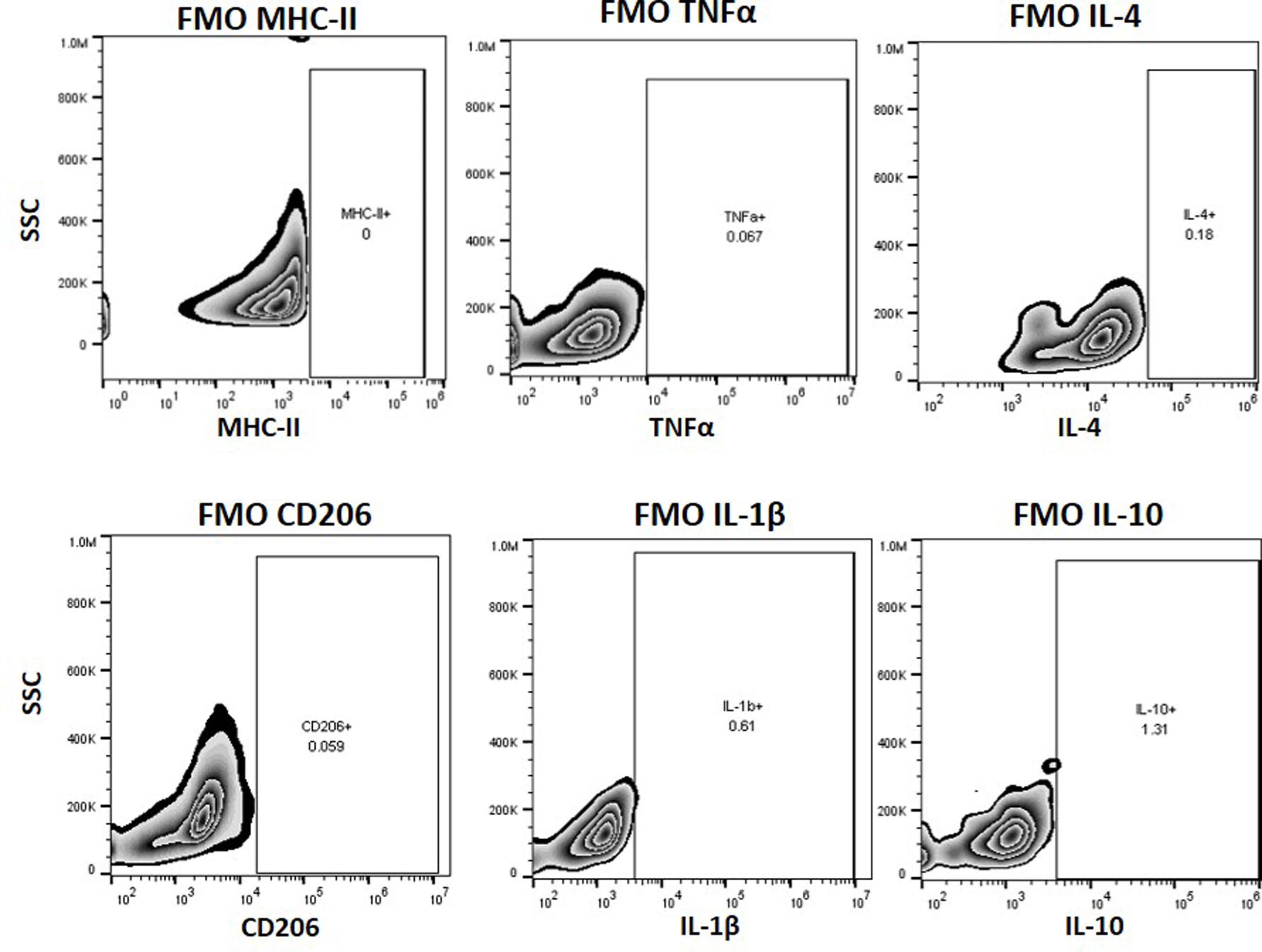

Supplement: Supplementary file 1 — Fig. S1 Cell type‐matched fluorescence minus one (FMO) controls for MHC‐II, TNF, IL‐4, CD206, IL‐1, and IL‐10. The boxed areas indicate the positive zone. [file EJN-47-140-s001.jpg]
