# Supplementary material for: Interferon regulatory factor 4/5 signaling impacts on microglial activation after ischemic stroke in mice
Source: Eur J Neurosci. 2018 Jan 16;47(2):140–9. doi: 10.1111/ejn.13778 (PMC5771847; doi:10.1111/ejn.13778)
Supplement: Supplementary file 2 [file EJN-47-140-s002.pdf]

EJN European Journal of Neuroscience FENS Federation of European Neuroscience Societies

Interferon regulatory factor 4/5 signaling impacts on microglial activation after ischemic stroke in mice

Abdullah Al Mamun, Anjali Chauhan, Haifu Yu, Yan Xu, Romana Sharmeen & Fudong Liu

Review timeline:

Submission date:	10 July 2017
Editorial Decision:	29 August 2017
Revision received:	03 October 2017
Accepted:	31 October 2017

Editor: Deniz Kirik

1st Editorial Decision 29 August 2017

Dear Dr. Liu,

Your manuscript was reviewed by external reviewers as well as by the Section Editor, Prof. Deniz Kirik, and ourselves.

The reviews collectively indicate that your experiments generated new and important information. However, there are several substantial issues that need to be clarified/resolved before we can consider your manuscript further for publication in EJN.

As you can see, both reviewers recognise that this is an interesting, well executed study but both raise some concerns that need to be addressed. Most of their points can be addressed by clarification of the text and the inclusion of additional analyses of the data. Please address each of their comments carefully.

In addition we noted the following points that need to be addressed.
- The title is not comprehensible to the general readership of EJN.
- Please reduce the number of abbreviations in the abstract
- Abbreviation list is incomplete.
- Please supply the full name of ethics committee that granted permission for the study.
- Include the dilutions of secondary antibodies used in immunohistochemistry.
- Include the total number of animals used.
- How were animals euthanised for flow cytometry experiments?
- Include the dilutions of antibodies used for flow cytometry.
- How were microglia sorted (the CytoFLEX machine doesn't have sorting functionality)?
- Include the dilutions of antibodies in the intracellular antibody cocktail?
- Centrifugation measured in rpm not g in some places (usage is not consistent)
- Please include more details for behavioural tests.
- Ensure that the reporting of statistical data adheres to EJN guidelines.
- Figures are rather small and hard to read, higher resolution versions are required.
- It is difficult to see the scale bars in fig 1
- Please replace all bar charts with much more informative scatter plots or similar (see our recent EJN editorial http://onlinelibrary.wiley.com/doi/10.1111/ejn.13400/full).

If you are able to respond fully to the points raised, we would be pleased to receive a revision of your paper within 12 weeks.

Thank you for submitting your work to EJN.

Kind regards,

Paul Bolam & John Foxe
co-Editors in Chief, EJN

EJN European Journal of Neuroscience FENS Federation of European Neuroscience Societies

Reviews:

Reviewer: 1 (Tomas Deierborg, Lund University, Sweden)

Comments to the Author
Generally, the paper is well-written and logically constructed. The experiment is conduct in proper way. However, there are still some gaps missing in the context I would like to point out.

Page 7: Explain the area in the penumbra that has been analysed. I would prefer to call it peri-infarct area since penumbra has a specific definition related to the salvation of tissue in the acute phase.

Fig1: Exactly where in relation to the infarct has the data been obtained?
The sham group seem to have more cells with IRF5 expression, seems like it IR in the neuropil compared to D3,10 and 30. Since the iba1/IRF5 cells are increased at D3 what cells are losing the expression? Since the number of IRF5 IR seems to be the same from Sham to D3. Relative expression of IRF5 would be interesting to see between groups.
Using other stainings e.g. for astrocytes or neurons to see if these cells are positive for IRF5/4 would be interesting.
If it is fold change, why is sham higher than 1?
Also, confocal pictures that show in detail the colocalisation would be appreciated.

Figure 3 and figure 4 don't have included 30-days old mice. However, the figure legends describe this time point. Please put the 30-day data in the figure or change the figure legends.
Demonstration of the control plots for FACS analysis, including MHC II and CD206, and intracellular stainings, TNF-a, IL-1beta, IL-4 and IL-10 are warranted. The gating of TNF-alpha and IL-4 is rather high.

Fig 5: D IL4 is high at 10d, which is not reflected by the microglia cell count in fig 4. What kind of cell could be responsible for the increase then?

Fig 6A: Please make a volumetric analysis.
Sometimes 3day and sometimes 3 day. Use space or be consistent.
Why behavioral tests? What do you want to compare with? The sham group should be presented in all the graphs.
This will only be interesting if you can correlate it with the microglia or cytokine data that you have obtained. By doing correlation analysis to see if the behavioral test correlate with the inflammatory alterations would make it possible to interpret something from the behavioral tests that otherwise are useless.
The following reference can be good to add when discussing the M1/M2 phenotype: PMID: 24669294

Reviewer: 2 (Midori Yenari, University of California- San Francisco, USA [assisted by Kota Kurisu])

Comments to the Author
This study investigated the dynamic reactions of microglial phenotype and its possible regulator, interferon regulatory factor (IRF) 4 and 5, in ischemic stroke model mice. The results demonstrated that post-ischemic dynamic changes in IRF 4/5`s balance seems modulate microglial phenotype (M1/2 polarization), as seen in peripheral macrophages. Experiments done in this study are well designed, and the manuscript is well-written. Furthermore, novel findings of this paper may provide us new knowledge which might be helpful to develop new therapeutic approaches for ischemic stroke. However, there are several issues authors should address to improve this paper for publication. A major weakness is that this is largely a descriptive and correlative study, but it does provide new insights into the post ischemic immune response.

Abstract: please clarify what is meant by 'sorted microglia.' Sorted for what?
For induction of transient MCAO, how did authors confirm occlusion of the MCA? The filament model was used, and it is well known that the blockage of blood flow at the origin of MCA is achieved blindly or 'by feel'. How did the investigators confirm that a stroke was achieved? Did authors evaluate post-stroke neurological deficit? Or, measure the cerebral blood flow?

How many animals were used in this study? And of these, how many animals were used for each analysis?? This should be noted not only in the figure legends, but also in the body of the manuscript.
For IHC analysis, authors noted that analysis was performed in the penumbral area. How then, did authors define the 'penumbra'?

In figure 1, the numbers of co-localized (IRF4/5 and Iba1) cells are expressed as fold changes. But what are these changed compared to?

The authors demonstrate that IRF 4 increases not only at day 10, but also at 24 hours after stroke (Figure

2). Is it also detectable in the IHC study? It might be better if authors can show IHC at 24 hours as well, to better delineate anatomical distribution and to corroborate flow cytometry data.

Authors conclude that the balance of IRF4 and 5 determine the phenotype of the microglia, because its time course change is almost same as that of M1/2 polarization. However, these studies are largely correlative and do not directly prove causation. Authors should acknowledge this in discussion section as the limitation.

Authors' Response 03 October 2017

Dear Editor,

Thank you very much for reviewing our submission titled "IRF5/4 regulatory axis balances microglial M1/M2 activation after ischemic stroke in mice". We thank all reviewers for their very constructive comments and suggestions, and have added new data and revised the manuscript accordingly (marked as red in the revised manuscript). Each reviewer's comments have been addressed below. We feel these changes have significantly strengthened this manuscript and hope that it will now be suitable for publication in "EJN"

COMMENTS FROM EDITOR

Your manuscript was reviewed by external reviewers as well as by the Section Editor, Prof. Deniz Kirik, and ourselves. The reviews collectively indicate that your experiments generated new and important information. However, there are several substantial issues that need to be clarified/resolved before we can consider your manuscript further for publication in EJN. As you can see, both reviewers recognize that this is an interesting, well executed study but both raise some concerns that need to be addressed. Most of their points can be addressed by clarification of the text and the inclusion of additional analyses of the data. Please address each of their comments carefully. On addition we noted the following points that need to be addressed.

We thank the editor for your comprehensive review. We have addressed each point sequentially below

(1) The title is not comprehensible to the general readership of EJN.

As suggested by the editor the manuscript title has been changed as "Interferon regulatory factor 4/5 signaling impacts on microglial activation after ischemic stroke in mice".

(2) Please reduce the number of abbreviations in the abstract

The number of abbreviations (i.e. MCAO, FC, and IHC) has been reduced in the abstract

(3) Abbreviation list is incomplete.

We have completed the list of abbreviations in the manuscript

(4) Please supply the full name of ethics committee that granted permission for the study.

Thank you very much for your kind concern. In the Methods part we have already put in the complete name of ethics committee.

(5) Include the dilutions of secondary antibodies used in immunohistochemistry.

We apologize for the lack of clarification. Now we have added the dilution information of the secondary antibody in the manuscript.

(6) Include the total number of animals used.

The total number of animals has now been added in the Methods part of the manuscript.

(7) How were animals euthanised for flow cytometry experiments?

For all experiment, the mice were euthonized by tribromoethanol (Avertin®) ip injection at a dose 0.25mg/g of body weight. Now, we have added this information in the Methods part.

(8) Include the dilutions of antibodies used for flow cytometry.

We have added all the dilutions of primary antibody in flow cytometry method section.

(9) How were microglia sorted (the CytoFLEX machine doesn't have sorting functionality)?

We apologize that we did not put sorting machine information in the manuscript, which has led to confusion. CytoFLEX machine was actually used for regular flow analysis. BD FACS ARIA II is the machine that we used for microglia sorting, and this information has been added in the Methods.

(10) Include the dilutions of antibodies in the intracellular antibody cocktail?

We have added all the dilutions of intracellular primary antibody in flow cytometry method section.

(11) Centrifugation measured in rpm not g in some places (usage is not consistent)

We have now used "g" instead of rpm throughout the manuscript.

(12) Please include more details for behavioral tests.

Thank you very much for your suggestion. We have now included the details of individual behavioral test in the manuscript.

(13) Ensure that the reporting of statistical data adheres to EJN guidelines.

We have cheeked the EJN guidelines for the reporting of statistical data and reported accordingly.

(14) Figures are rather small and hard to read, higher resolution versions are required.

We have now included the higher resolution images.

(15) It is difficult to see the scale bars in fig 1

We have resolve the scale bar issue.

(16) Please replace all bar charts with much more informative scatter plots or similar.

Thanks for the constructive suggestion. All the bar charts in figure 5 and 6 have been changed to scattered plots.

Reviewer: 1

Generally, the paper is well-written and logically constructed. The experiment is conduct in proper way. However, there are still some gaps missing in the context I would like to point out.

We thank the reviewer for his/her comprehensive review. We have addressed each point sequentially below:

(1) Page 7: Explain the area in the penumbra that has been analyzed. I would prefer to call it peri-infarct area since penumbra has a specific definition related to the salvation of tissue in the acute phase.

Answer: We agree with the reviewer that penumbra is actually related to the salvation of tissue in the acute phase of stroke. Since chronic time points (10d and 30d) were also involved in the study, we agree it's more appropriate to use "peri-infarct area" instead of penumbra. Thanks for this excellent point and we have changed the term throughout the manuscript.

(2) Fig1: Exactly where in relation to the infarct has the data been obtained?
The sham group seem to have more cells with IRF5 expression, seems like it IR in the neuropil compared to D3, 10 and 30. Since the iba1/IRF5 cells are increased at D3 what cells are losing the expression? Since the number of IRF5 IR seems to be the same from Sham to D3. Relative expression of IRF5 would be interesting to see between groups.
Using other staining's e.g. for astrocytes or neurons to see if these cells are positive for IRF5/4 would be interesting.
If it is fold change, why is sham higher than 1?
Also, confocal pictures that show in detail the colocalisation would be appreciated.

Answer: Thanks for pointing out this issue and we have now clarified the area (Figure 1A) where we imaged and quantified the positive cells. Image analysis of microglia/ macrophage markers (Iba1) and IRF4/5 were done in 8 ipsilateral regions (black boxes) at the peri-infarct area. Please see figure 1A.

We apologize for putting a wrong representative sham image in the manuscript. We have now replaced it with a correct representative iamge accordingly. The quantification of positive cells was presented as relative expression to sham and we have now clarified this in the Y-axis title and in the legend. We took the mean value of sham as calibrator and then normalized other group value. We have now corrected the fold change as 1 for IRF5+Iba1 double cells in Figure 1.

The expression of different subclass of IRF (IRFs) was initially asserted to be restricted to immune cells and melanocytic lineages. However, Guo et al. have reported that IRF4 is also expressed in neurons to function as a transcription activator of serum response factor (SRF) that is crucial to salvage neurons after stroke in mice (Guo *et al.*, 2014). In the present study, we also found that IRF4 expressed in neuron but not IRF5 (**See the figure below in this letter for the reviewer**). However, no expression of IRFs was found in astrocytes. Since the signaling of IRF4 in neurons is different than that in microglia, we decide to not include the data in the manuscript.

We have also tried to perform the confocal microscopy to show colocalization of IRF4/5 with Iba1 in the brain slices. Unfortunately, confocal microscopy in our 30 um coronal sections did not provide better colocalization due to high signal noise ratio. And we think the current immunofluorescence staining has sufficiently provided relevant information.

(3) Figure 3 and figure 4 don't have included 30-days old mice. However, the figure legends describe this time point. Please put the 30-day data in the figure or change the figure legends. Demonstration of the control plots for FACS analysis, including MHC II and CD206, and intracellular stainings, TNF-a, IL-1beta, IL-4 and IL-10 are warranted. The gating of TNF-alpha and IL-4 is rather high.

Answer: We apologize for the confusing information. We mistakenly mentioned 30-day data in the legends of Figure 3 and 4, which have now been corrected. For the demonstration of flow cytometry control plots, we have added all the FMO (i.e. MHC II and CD206, TNF-a, IL-1beta, IL-4 and IL-10) control plots in supplementary Figure 1 in the manuscript. We agree that the gating of TNF-alpha and IL-4 based on FMO is rather high compared to other surface marker (i.e: MHC II and CD206), probably due to the reason that after ischemic injury, microglia serve as the major source of production of anti- or pro-inflammatory cytokines depending on the cellular microenvironments.

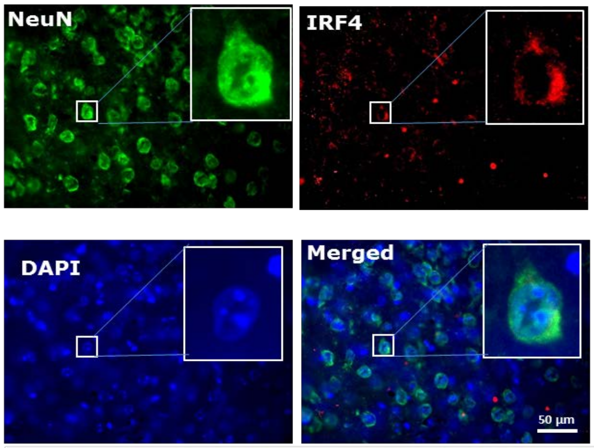

Figure 1 for reviewer: IRF4 expression in neuron at 3 days following 90 mins MCAO. Co-expression of IRF4 (red) with NeuN (green) in neuron were shown in upper and lower panel respectively. Scale bar 50µm.

(4) Fig 5: D IL4 is high at 10d, which is not reflected by the microglia cell count in fig 4. What kind of cell could be responsible for the increase then?

Answer: In Fig 5D, IL-4 level reflects the total brain level of this cytokine measured by ELISA; microglia, astrocytes, endothelial cells could all contribute to the level. In Fig4F we presented mean fluorescence intensity (MFI) of Il-4 and IL-10 on microglia. However, when we measured the IL-4 & IL-10 double positive microglia (Fig 4H), the result exhibited the similar pattern as that in Fig 5D. MFI of IL-4 on microglia, ELISA level of IL-4 in the brain, and IL-4+ microglia are three different parameters to evaluate cytokine expression and could have variability with each other. We think the data of IL-4 & IL-10 double positive microglia are more important because microglia phenotype is determined by expression of multiple cytokines other than one single cytokine.

(5) Fig 6A: Please make a volumetric analysis. Sometimes 3day and sometimes 3 day. Use space or be consistent.

EJN European Journal of Neuroscience FENS Federation of European Neuroscience Societies

Why behavioral tests? What do you want to compare with? The sham group should be presented in all the graphs.
This will only be interesting if you can correlate it with the microglia or cytokine data that you have obtained. By doing correlation analysis to see if the behavioral test correlate with the inflammatory alterations would make it possible to interpret something from the behavioral tests that otherwise are useless.
The following reference can be good to add when discussing the the M1/M2 phenotype: PMID: 24669294

Answer: Thank you very much for your concern. We did not show the volumetric analysis of the histological changes of the brains because the infarct is only evident at 24h and 3d after stroke; and at later time points (10d and 30d) the infarct is converted to cerebral atrophy due to the tissue loss. The structural dissimilarity makes it impossible to compare the infarct of 24h or 3d with the atrophy of 10d or 30d. However, behavior deficits can be compared at different time points and that's why we performed behavior tests. Since the time course data of histological change provide limited information and could confuse readers, in the revised manuscript we have removed the cresyl violet staining images.

We have fixed the issue of using either "3day" or "3 days". Throughout the manuscript we have used 3d instead of 3days or 3 days.

In the present study, we have performed a panel of behavior tests to evaluate the correlation of stroke outcomes with inflammatory responses at acute and chronic stages of ischemia. The data from IHC and flow cytometry suggested that there was a M1 polarization at 3d, and then the phenotype was switched to M2 at 10d. By behavior data, we found that the motor deficits assessed by corner test and neurological deficit score was significantly improved at 10d compared to 3d, suggesting that M2 microglial phenotype promotes neuronal survival and exert an endogenous effort to clean ischemic tissue debris and restrict brain damage.

We thank the reviewer's constructive suggestion and performed correlation test between cytokine expression and behavior deficits. We found the microglial expression of TNFα significantly correlated ($p=0.03$) with the neurological deficit score (NDS) at 3d and 10d**,** suggesting TNFα may play an important role in the inflammatory response. Our new correlation data has now been included in the manuscript as **Figure 6(D)**. In addition, behavior data of sham group have also been included in figure 6.

As suggested by the reviewer, the article "**PMID: 24669294"** has now been used as a reference while discussing M1/M2 phenotype in introduction section.

Reviewer: 2

Comments to the Author
This study investigated the dynamic reactions of microglial phenotype and its possible regulator, interferon regulatory factor (IRF) 4 and 5, in ischemic stroke model mice. The results demonstrated that post-ischemic dynamic changes in IRF 4/5`s balance seems modulate microglial phenotype (M1/2 polarization), as seen in peripheral macrophages. Experiments done in this study are well designed, and the manuscript is well-written. Furthermore, novel findings of this paper may provide us new knowledge which might be helpful to develop new therapeutic approaches for ischemic stroke. However, there are several issues authors should address to improve this paper for publication. A major weakness is that this is largely a descriptive and correlative study, but it does provide new insights into the post ischemic immune response.

Answer: We thank the reviewer for his/her enthusiasm for the manuscript. We agree that the present study is quite descriptive and mechanistic studies are warranted, which are on going in this lab.

(1) Abstract: please clarify what is meant by 'sorted microglia.' Sorted for what? For induction of transient MCAO, how did authors confirm occlusion of the MCA? The filament model was used, and it is well known that the blockage of blood flow at the origin of MCA is achieved blindly or 'by feel'. How did the investigators confirm that a stroke was achieved? Did authors evaluate post-stroke neurological deficit? Or, measure the cerebral blood flow?

Answer: We apologize for the lack of clarification of “'sorted microglia”. Microglia sorting is performed based on flow cytometry technique; we used BD FACS ARIA II because this instrument can precisely purify and separate immune cells based on cell surface markers from a mixture of total cell population. The gating strategy has shown in **figure 2A**. To measure the IRF4/5 mRNA expression, we collected sorted microglia after flow cytometry and subjected cells to mRNA extraction to perform RT-PCR.

Yes, in the present study, focal cerebral ischemia was produced by intraluminal occlusion of the left middle cerebral artery (MCA) for 90 minutes. It is true that surgeon can feel the resistance when the monofilament blocks the opening of MCAO. In addition to this, we routinely use laser Doppler flowmetry to measure the regional cerebral blood flow in all stroke animals. Animals that showed a regional cerebral blood flow reduction by at least 85% of baseline levels during MCAO were included for further experimentation. For further conformation, we have also performed neurological deficit scoring (NDS) in all the stroke animals. Now, this information has been included in the method section.

(2) How many animals were used in this study? And of these, how many animals were used for each analysis?? This should be noted not only in the figure legends, but also in the body of the manuscript.
For IHC analysis, authors noted that analysis was performed in the penumbral area. How then, did authors define the ‘penumbra’?

Answer: We thank the reviewer for his/her constructive comment. A total of 147 mice (55 sham-operated and 92 ischemic mice) were used in this study, including 8 mice that were excluded from further assessments because of either death after ischemia or failure in ischemia induction. Among them, a cohort of 6 stroke/4 sham animals was used for each experiment. This information has now been included in the revised manuscript.

The ‘penumbra’ is originally defined as the tissue immediately surrounding the infarct core in which there is reduced blood flow, impaired neuronal functionality, but preserved structural integrity; however, this area can evolve into infarct if reduced blood flow cannot be restored in a short time window. We recognize that the term “penumbra” is only applied to acute phase of stroke; both reviewers have brought up this important point (thank you) and we have now changed the term to “peri-infarct area” as “penumbra” does not exist in the chronic stage. We have now included Figure 1A to show the 8 different peri-infarct areas at the infarct boarder for our IHC analysis.

(3) In figure 1, the numbers of co-localized (IRF4/5 and Iba1) cells are expressed as fold changes. But what are these changed compared to?

Answer: In figure 1, all the quantification data were presented as relative changes compared with sham groups. We have now specified this in the legends.

(4) The authors demonstrate that IRF 4 increases not only at day 10, but also at 24 hours after stroke (Figure 2). Is it also detectable in the IHC study? It might be better if authors can show IHC at 24 hours as well, to better delineate anatomical distribution and to corroborate flow cytometry data.

Answer: We appreciate this constructive suggestion. We have now performed MCAO for a 24h IHC corhort to corroborate RT-PCR and flow cytometry data. Our new data demonstrated that IRFs were also detectable at 24h after reperfusion. However, there was no significance difference in $IRF4^{+}Iba1^{+}$ cell numbers between 24h and sham groups (Figure 1 in revised manuscript). The inconsistency of IRF4 protein expression in Fig.1 and IRF4 mRNA level in Fig. 2 at 24h may be due to the time difference between gene transcription and translation.

(5) Authors conclude that the balance of IRF4 and 5 determine the phenotype of the microglia, because its time course change is almost same as that of M1/2 polarization. However, these studies are largely correlative and do not directly prove causation. Authors should acknowledge this in discussion section as the limitation.

Answer: We agree with the reviewer that the present study is largely correlative and descriptive. But IRF5/4 signaling has never been reported in cerebral ischemic stroke; we think it is also important to report the relationship between IRF5/4 signaling and microglial response to stroke. Ongoing studies in the lab are

using genetic methods to mechanistically investigate the role of IRF5/4 signaling in mediating inflammatory responses after stroke. We have now acknowledged this limitation in the discussion part.

Again, we thank the editor and reviewers for their very insightful and helpful comments. We have added new data, revised the manuscript accordingly and provided a more thorough discussion that has strengthened our manuscript.

References:

Guo, S., Li, Z.Z., Jiang, D.S., Lu, Y.Y., Liu, Y., Gao, L., Zhang, S.M., Lei, H., Zhu, L.H., Zhang, X.D., Liu, D.P. & Li, H. (2014) IRF4 is a novel mediator for neuronal survival in ischaemic stroke. *Cell death and differentiation*, 21, 888-903.
